# Preliminary Assessment of Body Condition Score as a Possible Marker for the Targeted Selective Treatment of Dairy Sheep Against Gastrointestinal Nematodes

**DOI:** 10.1007/s11686-021-00470-9

**Published:** 2021-10-06

**Authors:** Claudia Tamponi, Giorgia Dessì, Antonio Varcasia, Stephane Knoll, Luisa Meloni, Antonio Scala

**Affiliations:** Parassitologia, Dipartimento di Medicina Veterinaria, Università degli Studi di Sassari, Sassari, Italy

**Keywords:** Body condition score, EPG, Mediterranean, Sheep, Targeted selective treatment

## Abstract

**Purpose:**

In the fight against anthelmintic resistance, targeted selective treatments (TSTs), where only a small percentage of a flock receives treatment, have become increasingly popular. Overall, implementation of such treatments can be based on various parameters including Body condition score (BCS). As infection with non-bloodsucking nematodes, frequently encountered on sheep farms in the central Mediterranean basin, commonly causes bodyweight reduction, the aim of this research is to evaluate the effectiveness of BCS as a parameter for the implementation of TSTs in lactating dairy sheep with subclinical gastrointestinal nematode (GIN) infections from the island of Sardinia, Italy.

**Methods:**

Faecal samples from 1012 ewes divided into 2 groups (third and fifth month of lactation) were collected and their BCS recorded. Faecal egg counts and coprocultures were performed for the assessment of the GIN burden and identification of present species.

**Results:**

An overall GIN prevalence of 85.4% with a mean eggs per gram (EPG) of faeces of 210.1 ± 347.3 was found. *Teladorsagia* spp. and *Trichostrongylus* spp. were the GIN genera most identified. Overall, animals with the lowest BCS had the highest EPG values and a negative correlation (*r* = − 0.163) between the EPG values and BCS of the studied animals was found, which was most significant for older sheep.

**Conclusion:**

This research confirmed BCSs and EPG values for GIN in sheep to be negatively correlated, particularly in older ewes. Application of TSTs for lactating sheep with a BCS < 2.25, especially to older ewes, could be beneficial in case of subclinical GIN infections, although further studies are needed to work out precise recommendation.

## Introduction

Gastrointestinal nematodes (GIN) represent a major health and zootechnical concern in small ruminant farming and may have significant negative effects on grazing dairy sheep [1–5].

Anthelmintic interventions are still the most frequently applied control measures against GIN infections [6] and in most cases, implementation of such treatments is done in a somewhat irrational fashion. In fact, anthelmintic treatment is often executed without adequate laboratory diagnostic support and active substances not always chosen wisely (e.g. based on cost effectiveness, by habit, etc.). Applied drugs are regularly selected without paying attention to the rotation of available molecule groups and/or with errors in posology [7–10]. Consequently, improper use of anthelmintics over the years has led to the onset and subsequent increase of anthelmintic resistance (AR) in GIN, bringing on significant negative repercussions for the control of these parasites, especially in some regions of the world [5, 11, 12].

The use of targeted selective treatments (TSTs) has become increasingly popular in the fight against AR in GIN and is aimed at reducing the number of animals treated within a flock. More specifically, TSTs entail solely the treatment of the most affected animals (usually a small percentage of the flock) [13, 14], thus leaving most of the animals in the flock untreated, ensuring the presence of larvae in *refugia* on pastures [15, 16]. Such larvae play a key role in AR prevention as this fraction of the parasite population will compete with resistant parasitic strains within treated animals, limiting their spread [15, 17].

Overall, TST programs can be based on various parameters such as: Faecal Egg Counts (FEC), Hematocrit (HCT) values, direct assessments of the degree of anemia through examination of the conjunctival mucosa (ocular mucosa coloration—OMC; FAMACHA method), faecal consistency score, milk production, weight loss and Body condition score (BCS) [2, 13, 18–23]. The choice of parameter varies according to the parasitological situation present (prevalent nematode species), type of production (meat, milk, or wool), available resources and technical circumstances of the farm [2, 24]. For example, FEC, employed with different threshold values, is an effective parameter but challenging when applied in large flocks [22] since egg excretion is dependent on the physiological situation of treated animals (e.g. after lambing, related to the post-parturient rise) and nematode species present (e.g. *Haemonchus contortus* females are particularly fecund, leading to high FECs even when worm burdens are low).

Dairy sheep rearing is widespread in particular regions of the Mediterranean basin, such as central-southern Italy and the Italian islands, where Sarda sheep is the most kept breed. More recently, Lacaune sheep and their crosses were introduced in the region as well [25].

Current scientific literature reports the presence of AR in GIN in the Mediterranean, although less severe compared to other sheep rearing regions (France, Spain, UK, Australia, New Zealand) [26]. Regardless, researching and validating new measures in the fight against AR (such as TSTs) is of significant economic-health interest, in particular in geographic areas where infections with bloodsucking nematodes such as *H. contortus* are widespread. Nonetheless, in that specific situation, the application of the FAMACHA method and the evaluation of HCT values may be considered most suitable for worm burden evaluation [22].

BCS is generally considered to be the simplest and cheapest indicator of an animal’s fat reserves for the use in periods of high-energy demand, stress and/or sub-optimal nutrition [27] as is for example characteristic of GIN infections [28, 29]. Especially, infections with *Trichostrongylus* spp., *Teladorsagia* spp. and other non-bloodsucking nematodes have been known to cause body weight reduction [30].

Previous studies have shown the use of BCS as a parameter for the implementation of TSTs to be very efficient. In this regard, solely sheep with a BCS ≤ 2 are treated as most animals with BCS > 2 can endure their GIN burden and thus do not require anthelmintic intervention [31]. Alternatively, some authors believe this treatment threshold should be more dynamic and adapted to the physiological (or productive) situation on a farm, similar to the use of FECs [32]. For instance, Aguirre-Serrano* et al*. [32] suggested treatment could be administered to sheep with a BCS < 3 specifically before mating and calving.

According to Calvete* et al*. [7], BCS could be used as a parameter for the implementation of TSTs in flocks of meat sheep with subclinical mixed GIN infections and would bring important benefits such as improved productivity and anthelmintic efficacy preservation.

In the lights of this, the aim of this study is to evaluate the effectiveness of BCS as a parameter for the implementation of TSTs in dairy sheep farms in the central Mediterranean basin where subclinical GIN infections with *T. circumcincta, Trichostrongylus* spp. and *H. contortus* are common. Accordingly, authors hope to contribute to AR reduction and help make dairy sheep farming more sustainable and profitable.

## Materials and Methods

### Animals and Management

The current study was conducted in 2019 on an experimental flock of Sardinian × Lacaune crossbreed milk sheep owned by the Sardinian Research Agency for Agriculture (AGRIS) located in Monastir (Sardinia—Italy, 39° 23′ 04.08″ N 9° 02′ 40.18″ E).

All sheep involved were identified through an endoruminal electronic bolus (detected with an automatic reader) and were subjected to the same diet consisting of 4 h of grazing on Alexandrian clover and Ryegrass meadow-pastures and supplemented with 600 g of Ryegrass hay and 600 g of concentrated feed.

Animals within this research were all lactating sheep and were divided into two groups. The first group was comprised of 394 ewes in their third month of lactation (February) and had an average milk production of 1.2 L/head per day. The second group was comprised of 618 ewes in their fifth month of lactation (April), with an average milk production of 1.1 L/head per day. Animals were subjected to two daily milkings using an automated milking machine. Lambs were separated from their mothers at about 1 month of age.

During the entirety of the research, no signs of clinical mastitis, diarrhea, respiratory symptoms or foot rot were noted within the flock.

### Sampling and Laboratory Analysis

Preliminary direct parasitological analysis was carried out 3 days prior to the main sampling. Post-mortem examination of four regularly slaughtered sheep (inspection of abdominal and thoracic viscera) and coprological examination of five pooled faecal samples composed of five individual samples each was performed. Pooled faecal samples were examined through sedimentation and flotation using a Zinc sulphate solution (1.35 specific gravity) and through the Baermann's method.

Within the entire research, faecal samples were collected directly from the rectum during the morning milking. Samples were placed in plastic containers on which the identification number of the animals were reported. Samples were transported in a cool box to the Laboratory of Parasitology and Parasitic Diseases of the Department of Veterinary Medicine of the University of Sassari (Sassari, Sardinia, Italy) for analysis.

Faecal samples collected during main sampling were individually analyzed through the McMaster's technique as described by Raynaud [33] using a supersaturated NaCl solution (1.2 s.g.) with an analytical sensitivity of 15 eggs/oocysts per grams (EPG/OPG) of faeces to obtain faecal egg count (FEC).

For each group, five pooled faecal samples were created from five individuals testing positive on quantitative analysis (McMaster) each. Following, coprocultures were prepared according to the method described by Euzeby [34]. Coprocultures were stored at 24 ± 1 °C for 14 days and cultured third-stage larvae (L3) isolated with a Baermann's apparatus. For each coproculture, at least 100 L3s were morphologically identified using morphometric keys described by van Wyk and Mayhew [35].

At the same time of faecal sampling, each animal was subjected to a BCS evaluation. Scoring of all sheep was carried out by the same veterinary researcher and was performed as indicated by Russel* et al*. [36] and Donoghue* et al*. [37]. Each animal was given a score ranging from 1 to 5 (1 = emaciated; 5 = obese) with a precision of 0.25.

### Data Collection and Processing

All data were collected on a spreadsheet (Microsoft Excel^®^) and subsequently processed using Epi Info™ v. 7.0 (Centers for Disease Control and Prevention [CDC]/World Health Organization [WHO], Atlanta, GA, USA), and Minitab^®^ v. 18.0 (Minitab, Inc., State College, Pennsylvania, USA) software. For the purposes of statistical processing, monitored sheep were divided into four groups according to age; (1) 1.5 years, (2) 2.5 years, (3) 3.5 years and (4) 4.5 years, and in six classes based on FEC; (1) negative, (2) ≤ 100 EPG, (3) > 100—≤ 300 EPG, (4) > 300—≤ 500 EPG, (5) > 500—≤ 1000 EPG, (6) > 1000 EPG. EPG data were transformed logarithmically [ln (*x* + 1)] as it showed a negative binomial distribution (most sheep showed low EPG values). Due to the fact data were not normally distributed (Kolmogorov–Smirnov test: *P* < 0.01), Spearman's correlation test, Mann–Whitney test and Kruskal–Wallis test were used to compare BCS in sheep stratified by EPG and age classes. Results with *P* values < 0.05 were considered statistically significant.

## Results

Preliminary parasitological examination revealed the presence of a single specimen of *Cysticercus tenuicollis* found in the mesentery, while copromicroscopic evaluation exclusively uncovered GIN eggs and a few *Eimeria* spp. oocysts.

For the 1012 faecal samples examined within the main analysis, an overall GIN prevalence of 85.4% (CI 95% 83.2–87.6) with a mean EPG value of 210.1 ± 347.3 was found. A prevalence of 92.1% (CI 95% 89.4–94.8) and 81.1% (CI 95% 78–84.2, *χ*^2^ = 266.82; *P* < 0.001), with mean EPG levels equal to 280 ± 424.9 and 165.6 ± 278.5 (Mann–Whitney test—*W* = 231,943.5; *P* < 0.001) were found for both groups, respectively (3 and 5 months of lactation).

Coprological analysis showed *Nematodirus* spp. eggs in 6.9% (CI 95% 4.8–9.8) and 4.5% (CI 95% 3.2–6.5) of the samples from the third and fifth month of lactation group, respectively. *Eimeria* spp. oocysts were found in 64.5% (CI 95 59.7–69.2) of the samples in the first group and in 63.9% (CI 95 60.1–67.7) in the second. OPG values were low (< 200 OPG) in both groups and thus not considered to affect BCS in this study.

Results of the coprocultures carried out for the identification of the GIN species present in both groups showed *Teladorsagia circumcincta* and *Trichostrongylus* spp. as the most prevalent nematodes (Table 1).

**Table 1:** Species composition of GIN identified by coprocultures

GIN species	Third month lactation group % L3 (n. L3)	Fifth month lactation group % L3 (n. L3)
*Teladorsagia* spp.	49.2% (62)	48.7% (55)
*Trichostrongylus* spp.	24.6% (31)	25.7% (29)
*Bunostomum* spp.	7.9% (10)	6.2% (7)
*Haemonchus* spp.	7.1% (9)	8.8% (10)
*Chabertia ovina*/*Oesophagostomum* spp.	5.6% (7)	6.2% (7)
*Cooperia* spp.	5.6% (7)	4.4% (28)

Average BCS values recorded in February were 2.833 ± 0.1765 and 2.822 ± 0.2661 in April. No significant difference was found between the BCSs in both groups (Mann–Whitney test; *W* = 205,547.5; *P* = 0.187).

Spearman’s correlation between the EPG values of each of the 1012 sheep monitored and their respective BCS was statistically significant (Rho = − 0.163; *P* = 0.000; *R*^2^ = 0.0222). The same correlation stratified according to month of lactation was equal to Rho = − 0.127; *P* = 0.012 and *R*^2^ = 0.0161 and Rho = − 0.207; *P* = 0.000 and *R*^2^ = 0.0428 in both groups, respectively.

Evaluation of the correlations between EPG and BCS values according to age groups was significant for sheep of 4.5 years of age in the third month of lactation group (Table 2) and for all sheep older than 2.5 years within the fifth month lactation group (Table 3).

**Table 2:** Correlations between EPG values and BCS scores according to age groups for sheep in their third month of lactation

N. sheep	Age class (years)	Spearman correlation	*P*	*R*^2a^
18	1.5	0.208	0.407	0.043
2^b^	2.5	–	–	–
174	3.5	− 0.095	0.231	0.009
200	4.5	− 0.192	0.006	0.037

^a^% shared variability

^b^Data not processed due to small number of animals

**Table 3:** Correlations between EPG values and BCS scores according to age groups for sheep in their fifth month of lactation

N. sheep	Age class (years)	Spearman correlation	*P*	*R*^2a^
131	1.5	− 0.064	0.469	0.004
149	2.5	− 0.189	0.021	0.036
176	3.5	− 0.165	0.028	0.027
162	4.5	− 0.207	0.008	0.043

^a^% shared variability

Analysis of mean BCS scores stratified according to the respective EPG classes, shown in Table 4, revealed an overall downward trend with the highest BCSs for coprologically negative animals and lowest BCSs for the animals with the highest EPG values (Kruskal–Wallis test: *H* value = 28.67; DF = 5; *P* = 0.000).

**Table 4:** BCS means stratified according to EPG classes

EPG Class	*N*	Mean	St. dev	95% CI
1	148	2.87	0.18	(2.83; 2.91)
2	378	2.83	0.18	(2.81.; 2.86)
3	281	2.81	0.19	(2.79; 2.84)
4	91	2.82	0.50	(2.78; 2.87)
5	76	2.74	0.19	(2.69; 2.80)
6	38	2.76	0.21	(2.69; 2.84)

Pooled st. dev = 0.2336

Parallel comparison (Mann–Whitney test) of mean BCSs of sheep stratified by EPG class also showed animals shedding less eggs to have significantly higher BCSs compared to animals with higher EPG values (Table 5). Mean BCSs of sheep within class 2 (≤ 100 EPG) were significantly higher than those from EPG classes 3 and 4 (*P* < 0.05) and borderline compared to those of EPG class 5 (*W* = 80,178.5; *P* = 0.053) (Table 5).

**Table 5:** Parallel comparison of BCS mean scores of sheep stratified by EPG classes (Mann–Whitney test)

	Mean BCS/EPG class 2	Mean BCS/EPG class 3	Mean BCS/EPG class 4	Mean BCS/EPG class 5	Mean BCS/EPG class 6
Mean BCS/EPG Class 1	*W* = 42,362.5 *P* = 0.032	*W* = 56,908.0 *P* = 0.004	*W* = 9073.0 *P* = 0.000	*W* = 6542.0 *P* = 0.000	*W* = 2697.0 *P* = 0.002
Mean BCS/EPG class 2	–	*W* = 127,466.5 *P* = 0.259	*W* = 91,517.0 *P* = 0.021	*W* = 89,531.0 *P* = 0.001	*W* = 80,178.5 *P* = 0.053
Mean BCS/EPG class 3	–	–	*W* = 15,658.0 *P* = 0.141	*W* = 52,357.5 *P* = 0.010	*W* = 45,695.5 *P* = 0.169
Mean BCS/EPG class 4	–	–	–	*W* = 7974.0 *P* = 0.290	*W* = 5980.0 *P* = 0.735
Mean BCS/EPG class 5	–	–	–	–	*W* = 2265.0 *P* = 0.633

GIN prevalence rates and mean EPG values in sheep stratified according to BCS are shown in Table 6.

**Table 6:** Prevalence rates of GIN and EPG mean values stratified according to BCS scores

BCS	Examined	Positives	Prevalence (%)	EPG mean	St. dev	95% CI
2.25	12	10	83.3	461.0	± 501.0	(266.0; 656.0)
2.50	114	106	93.0	289.1	± 451.9	(225.8; 352.4)
2.75	488	429	87.9	224.0	± 351.2	(193.4; 254.6)
3.00	364	291	79.9	164.9	± 297.6	(129.5; 200.3)
3.25	34	27	79.4	140.3	± 209.1	(24.4; 256.2)

Pooled st. dev = 344.464

Significant differences in mean EPG values stratified according to BCS were found through Kruskal–Wallis test (*H* = 27.84; DF = 4; *P* = 0.000).

## Discussion

The present study reports the monitoring of the relationship between FEC and BCSs of dairy sheep with subclinical GIN infections (a situation commonly encountered in flocks in the Mediterranean region) at 3 and 5 months of lactation to better understand the correlation between the two parameters.

Overall, the different degrees of GIN infection detected through FECs in the two investigated lactation groups did not significantly affect their respective BCSs. The average BCS at 3 months after lambing was slightly higher compared to that at 5 months, despite significantly higher EPG values (280 ± 424.9 at three months and 165.6 ± 278.5 at 5 months) and a marginally higher milk production.

The higher EPG levels at 3 months postpartum did not affect the animal’s BCS in a significant way as, most likely, these would be related to a final stage of the post-parturient rise phenomenon still present at the third month of lactation [29, 38] and thus not to represent a quantitative change in the actual number of adult GINs present nor their real pathogenic effect.

In any case, even with subclinical infections mainly caused by *Telardorsagia* spp. and *Trichostrongylus* spp. and characterized by an EPG mean of about 200, significant negative correlations between BCS and EPG levels in both lactation groups were found, although with fairly low R2 values (explained variance) (< 2%). Furthermore, such negative correlations were most evident in sheep from older age classes (> 3.5 years) in which the explained variance values (R2) were slightly higher (about 4%).

In addition, the finding of the non-bloodsucking nematodes *Teladorsagia circumcincta* and *Trichostrongylus* spp. as the more prevalent species in the present study, also reported among the prevalent genera in Europe, confirms the applicability of BCS as a parameter for the implementation of TSTs in the area as these worms are recognized to affect weight gain in sheep. A ratio between weight gain in infected and weight gain in control animals of 0.81 and 0.78, respectively, has been described [4].

Overall, average BCSs showed a significant decrease with increasing EPG classes, serving as further confirmation of the negative correlation between BCS and EPG of GIN in sheep. Regardless, for dairy sheep under the specific conditions within this research, BCS assessment was not found to be a sufficiently precise enough marker for the implementation of TSTs.

Furthermore, it is entirely plausible that the GIN infestation intensity detected within this study does not have any significant influence on the production performance of Sarda × Lacaune dairy sheep.

On the other hand, 13.3% of the monitored sheep within this research were classified into EPG classes 5 and 6, i.e. with values > 500 UPG, and thus should be treated with anthelmintics as indicated by Ambrosi [39].

In conclusion, the results obtained in the present study seem to confirm that, in general, all sheep with BCSs > 2 to have a GIN burden compatible with production [31], although application of TST for sheep with a BCS < 2.25, especially the older ewes, could lead to a significant reduction of the GIN egg contamination on pasture.

Although still preliminary, this study showed that in crossbred Sarda × Lacaune dairy sheep with subclinical GIN infestations at 3 and 5 months postpartum, above all, older sheep would need to be treated as a stronger negative correlation between BCS and EPG was shown for these animals. It would be beneficial to repeat the present study under alternative circumstances in the future, such as in growing animals or dairy sheep under highly parasitic conditions to reach definitive conclusions on the matter.
